# Green synthesis of peptide functionalized reduced graphene oxide (rGO) nano bioconjugate with enhanced antibacterial activity

**DOI:** 10.1038/s41598-020-66230-3

**Published:** 2020-06-10

**Authors:** Shubhi Joshi, Ruby Siddiqui, Pratibha Sharma, Rajesh Kumar, Gaurav Verma, Avneet Saini

**Affiliations:** 1 Energy Research Centre, Panjab University, Chandigarh, U.T. 160014 India; 2 Department of Biophysics, Panjab University, Chandigarh, U.T 160014 India; 3 Department of Physics, Panjab University, Chandigarh, U.T. 160014 India; 4 Dr. Shanti Swarup Bhatnagar University Institute of Chemical Engineering & Technology, Panjab University, Chandigarh, U.T. 160014 India; 5 Centre for Nanoscience and Nanotechnology (U.I.E.A.S.T), Panjab University, Chandigarh, U.T. 160014 India

**Keywords:** Biophysics, Microbiology, Materials science, Nanoscience and technology

## Abstract

Bioengineered nanoconjugates have enormous potential as a multifunctional platform for biomedical applications. Conjugation between biotic and abiotic materials enables formulation of nanoconjugates with enhanced physico-chemical properties, increased stability and ability to overcome the inherent shortcomings of individual materials. In this study, we report the preparation and biophysical characterization of an antibacterial system formulated by functionalizing reduced graphene oxide (rGO) with an antimicrobial peptide via covalent as well as non-covalent interaction mechanisms. Environmentally benign synthesis approach was adopted for the formation of rGO, using L-ascorbic acid as a reducing agent. Covalently conjugated peptide-graphitic conjugate displayed improved antibacterial efficacy against *Escherichia coli* with considerably low cytotoxic activity towards erythrocytes in comparison to self-assembled conjugate and rGO alone. The studies described herein are highly significant in the field of biomaterials and aims to open new avenues of research focusing on a plethora of applications as a prospective non-toxic substitute to conventional antibacterial approaches.

## Introduction

Graphene-based materials like graphite, graphene oxide (GO) and rGO (reduced graphene oxide) have been explored for numerous biomedical applications ranging from diagnostics to delivery of therapeutics owing to their inimitable physiochemical characteristics, renewability and economical raw material procurement^1,2^. Amongst these, oxygen-rich GO exhibits far ranging applications owing to occurrence of epoxide, hydroxyl, and carboxylic moieties in its structure. In comparison to GO, rGO lacks sufficient reaction sites and functional groups that limit its applicability^3^. Although, rGO has been reportedly used in construction of sensors, but it has not been explored much for its therapeutic efficacy^4,5^. Thermal annealing along with application of reducing agents are used to eliminate functional groups usually present on GO to produce rGO^6,7^. Over the last few years many methods for preparation of rGO have been reported but most of them are time consuming, use toxic reagents and produce a low yield^8^. In order to reduce the harmful effects of these reducing agents’ efforts are being focussed on using naturally derived agents which are non-toxic. Therefore, it is necessary to opt for environment friendly reducing agents like L-ascorbic acid which give a better yield as compared to the conventional reducing agents^9,10^. Although reduction of GO by application of L-ascorbic acid has been reported previously, but functionalizing it with biomolecules like peptides has not been reported earlier^11^.

**Figure 1:**
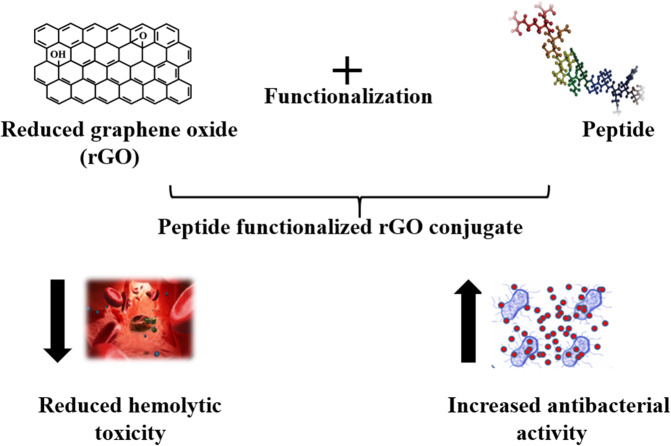
Schematic representation of a bioengineered peptide functionalized rGO conjugate with reduced hemolytic toxicity and increased antibacterial efficacy. This image was produced using Microsoft PowerPoint (https://products.office.com/en-in/powerpoint).

Antimicrobial peptides (AMPs) are essential components of the innate immune system^12^. They are widely distributed amongst a wide variety of life forms ranging from microorganisms to humans. AMPs display antibacterial function by interacting with the surface of the cell membrane thereby causing disintegration of lipid bilayer present on the bacterial structure followed by cell death^13^. AMPs are lucrative substitutes for traditional antibacterial agents because of their specificity, broad spectrum activity against microorganisms and lower susceptibility towards resistance development^13^. However, the applicability of AMPs is limited by challenges related to small contact surface area, hemolytic toxicity and low enzymatic stability^14^. Amongst the wide variety of antibacterial peptides, Chicken cathelicidins have been reported to effectively kill a variety of bacterial species inducing little or no resistance^15,16^. In this study we have selected, the N-terminal fragment of Cathelicidin-2 (CATH-2) as the AMP for conjugation to rGO, because of its reported broad-spectrum activity against various pathogenic microorganisms (Fig. 1)^17,18^.

**Figure 2:**
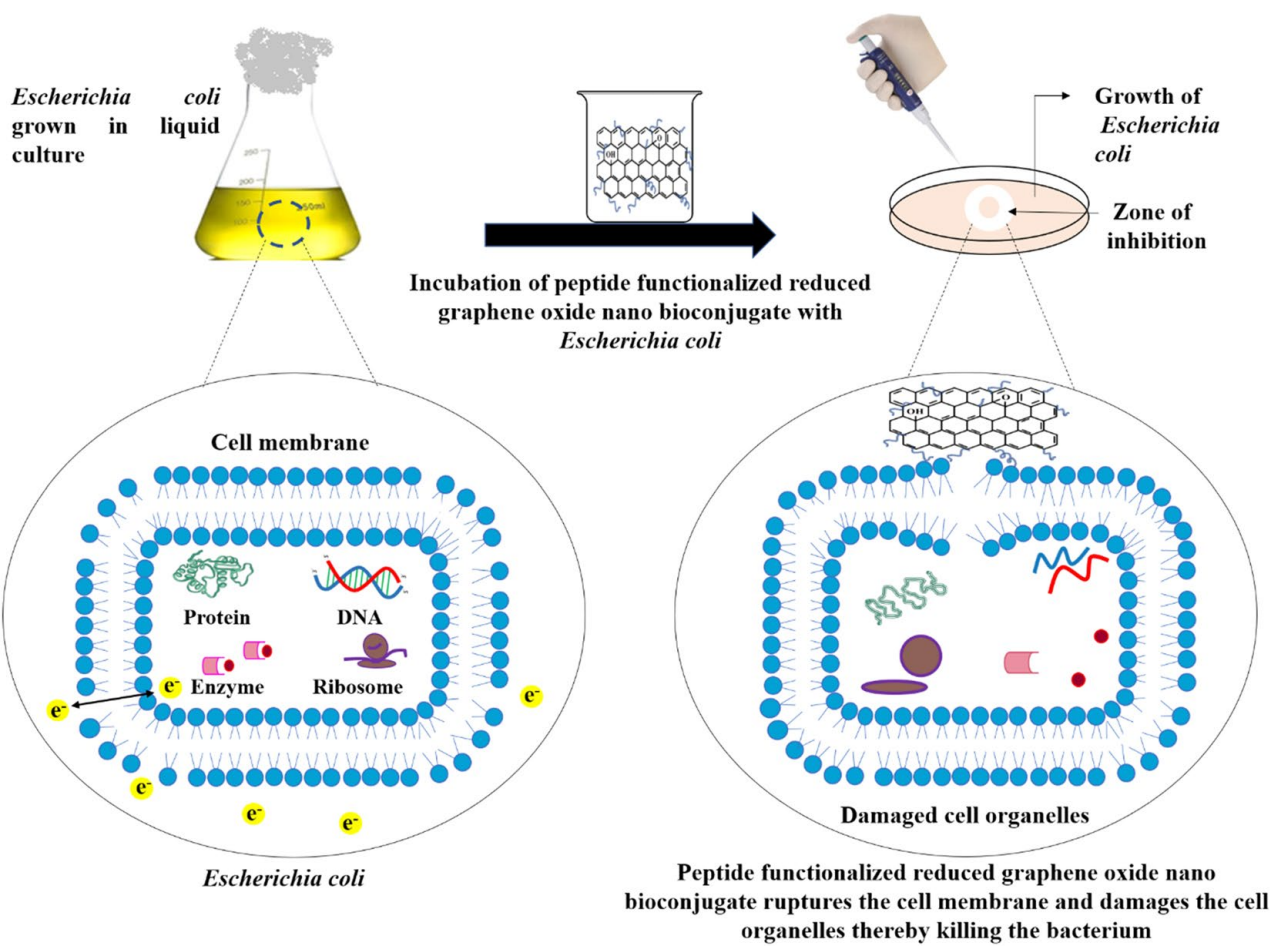
Schematic illustration depicting antibacterial action of peptide functionalized reduced graphene oxide (rGO) nano bioconjugate. This image was produced using Microsoft PowerPoint (https://products.office.com/en-in/powerpoint).

Conjugation of AMPs with nanoparticles has been proposed to result in increasing the local concentration of the peptide at the site of delivery, thereby increasing the efficacy^3,19^. The probable mechanism engaged in antibacterial activity exhibited by nano bioconjugates can be accredited to membrane stress leading to cell membrane degradation which further results in loss of membrane integrity causing outflow of cell organelles, leading to cell lysis (Fig. 2)^20,21^.

Numerous peptide-nanoparticle systems formed by application of harsh and toxic reagents have been explored as antimicrobial agents. Such complexes comprise of residual toxic substances and are usually not appropriate for biomedical based applications^22^. However, antibacterial peptide functionalized nanoparticle systems formulated via green route have not been prominently explored.

Motivated by this, we used different approaches to couple the antimicrobial peptide CATH-2 with rGO by covalent and non-covalent interaction mechanism. Covalent interaction between peptide and rGO was established by use of biocompatible crosslinkers; whereas, non-covalent interaction was facilitated by a facile self-assembly approach. Biophysical characterization techniques provided insight into the interaction behaviour and structural properties of the formed nano bioconjugates. The formed conjugates were evaluated for their efficiency to inhibit the growth of *E. coli*, a gram-negative bacterium. Further, the hemolytic cytotoxicity of the conjugates was also evaluated.

To the best of our knowledge, no study regarding the application of peptide functionalized rGO nano bioconjugate as an effective antibacterial agent has been reported. With further developments, we aim to expand the application of the reported nano bioconjugate as a prospective substitute to conventional antimicrobial approaches.

## Results

### Synthesis and characterization of L-ascorbic acid reduced graphene oxide

**Figure 3:**
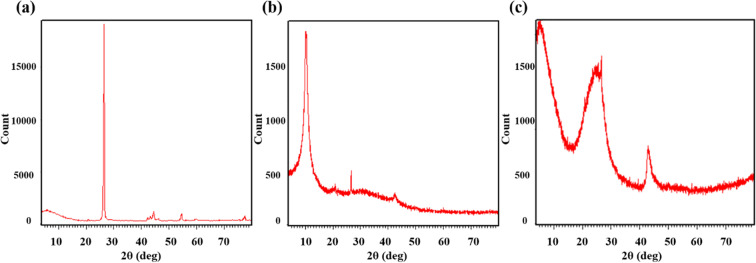
XRD spectra of graphite (**a**), GO (**b**) and rGO (**c**).

L-ascorbic acid was used as a reducing agent to produce rGO from graphite. It is a mild and nontoxic alternative for conventional reducing agents. Moreover, it can be used for large scale production of rGO. L-ascorbic acid has a tendency to maintain its reducing functionality in acidic as well as alkaline pH. This property aids in converting the residual Mn(VII) ions to a more soluble Mn(II) ions, thereby reducing the potentially hydrophilic, oxygen functionalities containing GO to rGO during the reduction process^11,23^. The crystallographic structure of graphite nano powder, GO and rGO was analysed by conducting X-ray diffraction (XRD) studies. Figure 3(a–c) illustrates XRD patterns of graphite, GO and rGO, respectively.

Graphite shows a single sharp characteristic peak at 26.7° corresponding to the d-spacing with interlayer distance calculated to be 0.34 nm. Whereas, upon reduction by L-ascorbic acid, the graphite peak shifts and gives a broad band centred at 24° and an increased d-spacing of about 0.37 nm. Occurrence of a single peak in graphite scan represents presence of robust interlayer covalent bonds within the carbon layers and existence of weak Van der Waals interactions amid consecutive carbon sheets. After reduction, broadening and shifting of the characteristic diffraction peak of graphite from 26.7° to 24° is attributed to the short-range order in randomly arranged stacks along with removal of some oxygen-containing functional groups^24^. This is an indication of graphitic network restoration in rGO by the elimination of oxygen moieties.

Another less intense peak observed at 42° corresponds to occurrence of turbostratic carbon assembly; along with structural defects in rGO, this peak is frequently observed in materials of graphitic origin^25,26^. A small characteristic diffraction peak of graphite oxide at approximately 2θ = 9° remained in the XRD pattern of rGO depicting incomplete reduction of oxygen-containing functional groups. In rGO, d-spacing changed due to the removal of few oxygen containing functional groups which indicates re-establishment of sp^2^ network on reduction, composed of free-standing graphene sheets^27^. The interlayer space of rGO is larger than that of pristine graphite, due to the presence of complicated residual contributions of intercalated water molecules between the layers. In comparison to XRD pattern of graphite, rGO displayed an amorphous structure because of decomposition of oxygen-containing groups which also eliminates carbon atoms from the carbon plane resulting in lattice defects^24^. These X-ray diffractograms demonstrate successful reduction of graphite nanoparticles to yield a randomly arranged reduced graphitic compound possessing an exfoliated structure which can prove to be beneficial during conjugation studies.

### Spectroscopic characterization of rGO and peptide functionalized rGO-nano bioconjugates

**Figure 4:**
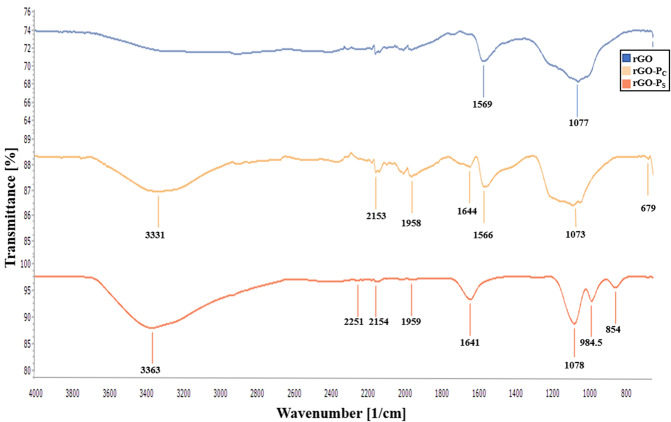
FT-IR spectra of rGO (blue line), rGO-P_C_ (orange line), rGO-P_S_ (red line).

FT-IR spectroscopy of rGO (blue line), rGO-P_C_ (orange line) and rGO-P_S_ (red line) is shown in Fig. 4. In FT-IR spectroscopy of rGO, the absorption peaks observed at 1569 and 1077 cm^−1^ can be attributed to C=C alkenes bending confirming the formation and recovery of sp^2^ carbon structure of rGO and presence of C-O group, respectively. The discussed rGO peaks show reduction of oxygen-containing groups by the action of reducing agents. The covalent bonding of peptide with rGO was confirmed by the presence of absorption bands at 3331, 2153, 1958, 1644, 1566, 1073 and 679 cm^−1^. The broad peak at 3331 cm^−1^ represents N-H stretching due to secondary amide groups. The weak intensity peak at 2153 cm^−1^ corresponds to the characteristic peak of C≡C alkynes. Strong peaks ranging from 1644 to 1580 cm^−1^ attributes to in-plane NH bending vibration from the peptide corresponding to amide bond^27^. Moreover, the representative peak of C-H binding at 1958 cm^−1^ is also exhibited in rGO-peptide (rGO-P_C_) spectrum, which confirms covalent binding of peptide with rGO^28^. The peak at 679 cm^−1^ is ascribed to N-H bending vibrations of amine.

Spectra of rGO-P_S_ exhibits peaks at 3363, 2251, 2154, 1959, 1641, 1078, 984.5, 854 cm^−1^. The strong band at 3363 cm^−1^ corresponds to N-H stretch of aliphatic primary amines. The presence of C-O alcohol group was revealed from the peak at 1078 cm^−1^. The spectrum around 2154 cm^−1^ attributes to C≡C alkynes. Similar peaks of amide bond around 3400 cm^−1^ and 1600 cm^−1^ in both the conjugates confirm absorption of peptide^28^. In the rGO conjugates, the O-H vibrations diminished whereas the carbonyl C=O peaks appeared, but with weaker intensities. The strong IR peak observed near 2300 cm^−1^ is due to the -NCO vibration which clearly indicates the formation of amides from peptide/reduced graphene oxide COOH group^27^. The FT-IR spectra of the peptide-rGO complex displays modification of rGO by peptides.

The presence of elements carbon (C), oxygen (O), nitrogen (N) and sulphur (S) was identified in the synthesized rGO-peptide nano bioconjugates (rGO-P_S_ and rGO-P_C_) by energy-dispersive X-ray (EDX) spectroscopy (Table 1). Presence of higher percentage of oxygen along with low carbon percentage was observed in rGO as compared to graphite.

**Table 1:** Comparison between weight percentage of different elements present in graphite, rGO, rGO-P_C_ and rGO-P_S_.

Element	Graphite Weight% Atomic%	rGO Weight% Atomic%	rGO-P_C_ Weight % Atomic %	rGO-P_S_ Weight% Atomic%
C	96.65; 32.89	82.72; 41.73	75.73; 23.58	59.54; 38.35
O	2.89; 2.87	17.28; 22.56	19.14; 9.41	35.16; 38.94
N	—	—	2.08; 0.18	0.19; 0.39
S	0.04; 0.10	—	2.08; 0.18	0.19; 0.39

**Figure 5:**
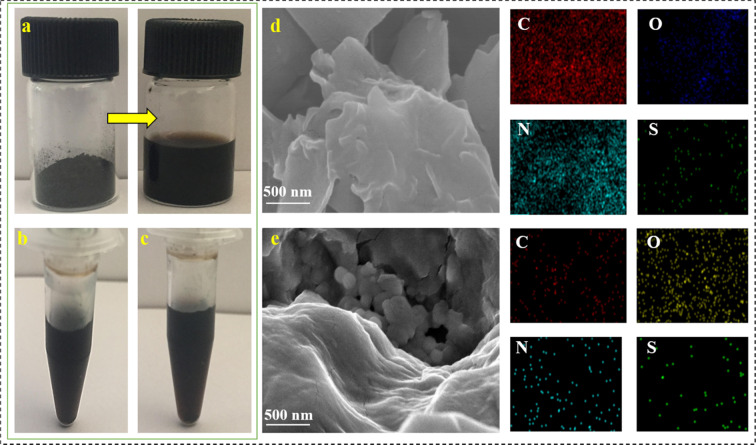
(**a**) Images depicting rGO powder before (left) and after dispersion in de-ionized water (right) (**b**) rGO-P_C_ (**c**) rGO-P_S_ (**d**) FESEM characterization of rGO-P_C_ along with elemental mapping showing carbon, oxygen, nitrogen and sulfur (**e**) FESEM characterization of rGO-P_C_ along with elemental mapping.

Increase in percent oxygen content was observed in both conjugates i.e., rGO-P_C_ and rGO-P_S_. Figure 5 depicts the mapping results of the conjugates revealing presence of other elements such as nitrogen and sulphur. The increased oxygen content of the conjugates can be attributed to the side chains of the various amino acids of the peptide.

### Microscopic characterization of rGO and peptide functionalized rGO-nano bioconjugates

**Figure 6:**
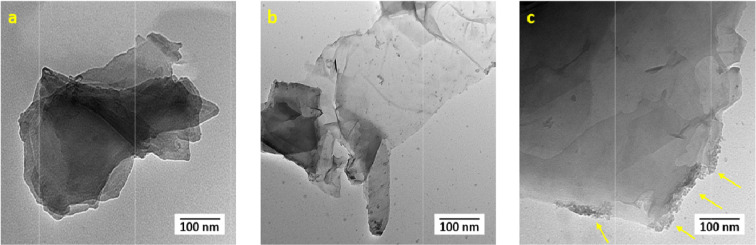
TEM micrographs of rGO (**a**), rGO-P_C_ (**b**) and rGO-P_S_ (**c**).

Transmission Electron Microscopy (TEM) was used to measure the particle size and investigate the morphology of the samples; Fig. 6 reveals rGO structure before conjugation (**a**), rGO-P_C_ (**b**) and rGO-P_S_ (**c**), respectively at 100 nm magnification. TEM micrograph of rGO reveals a transparent layered rGO with few wrinkles on its surface. The edges of the sheet appeared to be smooth. rGO mostly consists of exfoliated, few-layered sheets, that are wide and flat. Further, TEM images reveal that the oxidation-reduction process leaves disordered carbon inclusions within the sheets depicting successful reduction of graphite to rGO. The exfoliated edges of rGO sheets act as binding sites for peptides^29^. The TEM images of rGO-P_C_ shows the presence of a dense network of peptide on the surface of rGO sheets. This changed the morphology and thickness of rGO sheets confirming the covalent conjugation of peptide on rGO sheets. Abundant peptide fibrils filled the entire field of vision of rGO surface.

Studies show that rGO can be functionalized by covalent attachment of atoms or molecular groups to sp^2^ carbons. Such modifications maintain the 2-D lattice of rGO, however, due to the loss of the π-conjugated electron cloud present above and below the rGO plane, there are dramatic changes to its properties. rGO lends itself to covalent functionalization due to the presence of defects in the graphene lattice that act as reaction sites^30^. TEM images of rGO-P_S_ reveal abundant deposition of peptides mainly at the edges of the rGO flakes. A high-resolution scan of the complex tip was analysed and thickness was observed to be significantly larger than the thickness measured for isolated rGO flakes. The anchoring force that enables the binding of peptides on the edges or planar rGO surface can be attributed to electrostatic or π interactions. Molecules that can be tailored to recognize and interact either on the planar surface or edges of rGO can prove to be beneficial in modulating properties of nanoconjugates^31^. The size of rGO and peptide was found to be 16 nm and 7 nm, respectively. Significant changes were observed in the size after formation of the conjugates. The conjugate formed by self-assembly was observed to be 24 nm in size with 18 peptides bound to an individual sheet of rGO. This calculation was done using TEM images by a protocol^32^, already described in the literature.

### Water contact angle measurement

**Figure 7:**
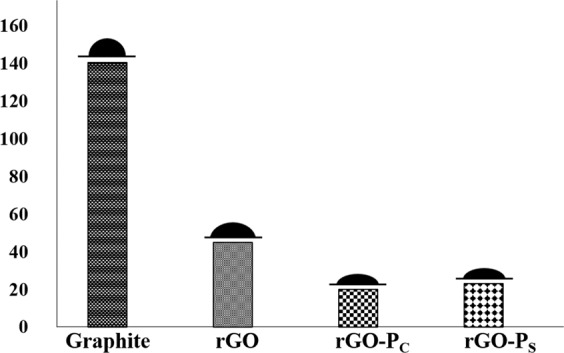
Graphical representation of WCA displayed by Graphite, rGO and rGO peptide conjugates (rGO-P_C_ and rGO-P_S_).

Wettability of a nanocomposite is an important parameter to study the interaction between water molecules and material surface. Wettability and adhesion analysis are useful in regulating the nanostructure and chemical configuration of nanomaterials^33^. The water contact angle (WCA) obtained between water droplet and Graphite, rGO, rGO-P_C_ and rGO-P_S_ are 140°, 44°, 20° and 23°, respectively (Fig. 7).

Graphite exhibited highest WCA at 140° amongst the samples being evaluated. A material is said to be hydrophilic when it exhibits a WCA of less than 90° and hydrophobic in case the angle is more than 90°. Therefore, it can be stated that graphite is hydrophobic in nature. The hydrophobicity exhibited by graphite can be attributed to numerous reasons like lack of polarity due to presence of carbon atoms in its structure and adsorption of hydrocarbons from air which causes decrease in the surface energy of the substrate thereby increasing its hydrophobicity. High contact angle value also indicates smoothness in graphite’s topology^34,35^. A decrease in hydrophobic behaviour was displayed by rGO (44°) synthesized from graphite nanopowder. It can be associated to high degree of reduction leading to removal of oxygen thereby enhancing the corrugation on its surface. Conjugation of rGO with peptide caused considerable increase in wettability from 44° to 20° in rGO-P_C_ and 23° in rGO-P_S_. Both the prepared conjugates are observed to be hydrophilic in nature due to addition of negatively charged functional groups present on the rGO-peptide along with presence of Van-der Waals interactions between the conjugate^36^. Numerous factors affect the WCA in graphitic nanocomposites like surface morphology, number of layers present, temperature of thermal treatment, method employed for sample deposition and environmental factors like relative humidity^37,38^. Understanding of the wettability characteristics of graphene-based nanocomposites provides direction to the research towards specific biomedical applications.

### Antibacterial activity

**Figure 8:**
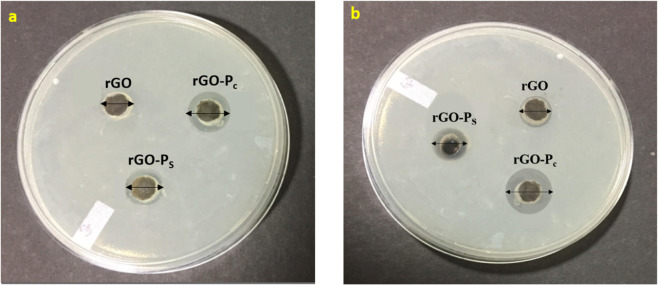
Inhibition zone of rGO, rGO-P_C_ and rGO-P_S_ on *E. coli* using well diffusion method at (**a**) 125 µg/mL and (**b**) 250 µg/mL.

Antibacterial activity of rGO, peptide and peptide conjugated rGO complex (rGO-P_C_, rGO-P_S_) was evaluated against *Escherichia coli* (*E. coli)* as a model bacterium since it is the most prevalent gram-negative infection-causing agent in humans^39^. The concentration dependent bacterial inhibitory activity of GO and antimicrobial peptide was studied by performing MIC (Minimum Inhibitory Concentration) method. MIC is interpreted as the required minimum inhibitory concentration for bacterial membrane disruption. The samples were incubated with *E. coli* cells at different concentrations ranging from 500–7.6 µg/ml for 16 hours. The MIC of GO and antimicrobial peptide was found to be 125 and 64 μg/mL, respectively. Further, agar well diffusion method was performed to evaluate the zone of inhibition formed by rGO and the synthesized conjugates. As displayed in Fig. 8, rGO-P_C_ exhibited a higher antibacterial activity followed by rGO-P_S_. At 125 µg/mL the zone of inhibition diameter for rGO, rGO-P_C_ and rGO-P_S_ was observed to be 11 mm, 15 mm and 13.2 mm. At 250 µg/mL a clear zone surrounding the sample wells was observed to be 13.27 mm, 21.81 mm and 16.50 mm of rGO, rGO-P_C_ and rGO-P_S,_ respectively.

The occurrence of clear zone is an indicator of antibacterial activity of the samples against *E. coli*. The zone of inhibition is more for rGO-P_C_, indicating a higher inhibitory efficacy, followed by rGO-P_S_ in comparison to rGO. rGO structure comprises of sharp edges called “nano-knives”. Upon interaction, these nano-knives disrupt the bacterial membrane thereby causing DNA leakage and consequently death of the pathogen^20^. It can be inferred that conjugation of peptides with rGO, imparted antibacterial property to the graphitic material. The difference in the antibacterial activity of the two peptide conjugates lies in the peptide conjugation process and the mode of action of antimicobial peptides. AMPs kill bacteria by disrupting the cell membrane through barrel stave model or toroidal model^40^. In this mechanism of action the amino acid side chain plays a very importat role. The cationic side chains aid in the interaction of the peptide with negatively charged phospholipids of the bacterial membrane and hydrophobic and aromatic side chains help in disruption of the membrane, causing increased permeability leading to leakage of cell organelles thereby causing death of the bacteria^41^. When we form a conjugate between peptide-rGO through covalent modification method the side chain functionalities are free to carry out their usual role of bacterial membrane disruption.Whereas, in self-assembly conjugation the peptide adheres to the rGO surface through non-covalent interactions with the various side chains, thereby making them unavailable to carry out their function of membrane disruption hence explaining the reported increase in antibacterial activity of the conjugate formed by covalent interaction method. As a matter of fact, surface neutralisation of the bacterial membrane is an important physicochemical parameter governing the antibacterial activity of potential antibacterial conjugates which come directly in contact with the bacterial surface. Previous studies have reported that untreated *E. coli* exhibits a negative zeta potential usually in the range of −23.0 to −44.0 to mV^42,43^. Whereas, both antimicrobial peptides and rGO, possess a positive zeta potential value, thereby having the capability to neutralize negative charge on *E. coli* surface and exhibit bactericidal activity^44,45^.

### *In-vitro* Hemolytic activity

**Figure 9:**
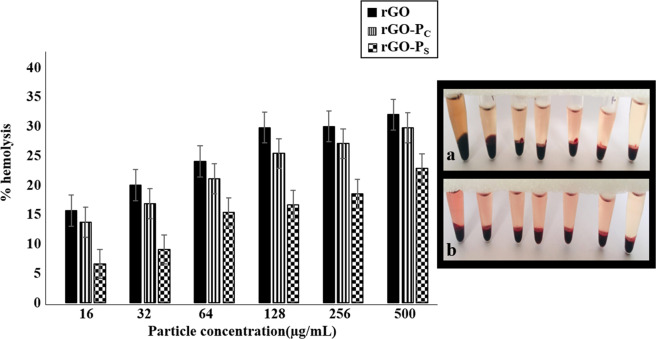
Percent hemolysis of RBCs incubated with increasing concentrations (16 to 500 μg/mL of rGO, rGO-P_C_ (**a**), rGO-P_S_ (**b**) for 3 hours at 37 °C (n = 3).

To evaluate the efficacy as a therapeutic agent the hemolytic activity of rGO, rGO-P_C_ and rGO-P_S_ was determined by measuring their effect on blood samples (Fig. 9). The compromise of RBC membrane by rGO and the formed conjugates was studied in a dose dependent manner.

*In-vitro* hemolytic assay enabled quantification of hemoglobin release as a result of RBC (Red blood cell) membrane lysis. It was observed that, both the conjugates exhibited a negligible hemolytic activity as compared to rGO alone. rGO-P_C_ displayed a significantly lower hemolysis percentage as compared to rGO-P_S_. The maximum hemolysis value was observed to be less than 22% at a concentration of 500 µg/mL by rGO-P_C_ conjugate whereas, at same concentration rGO-P_S_ exhibited 28% cell lysis. This may be attributed to the fact that the peptides self-assembled at the edges of rGO leaving its surface exposed to interact and disrupt the RBCs more readily^46^. On the other hand, covalently attached peptides mask the electrostatic moieties present on the surface of rGO. Hence, it was observed that the hemolytic cytotoxicity caused by rGO was higher in comparison to peptide whereas, rGO-P_C_ displayed a lower hemolytic cytotoxicity in comparison to the self-assembled conjugate.

## Discussion

A sustainable peptide functionalised rGO conjugate system with a potential to exhibit reduced cytotoxicity and enhanced antibacterial activity was successfully synthesized through application of green chemistry approach. The characterization of the nanoconjugate uses XRD, FT-IR, EDX, TEM and WCA techniques. It was observed that corrugations present on rGO lend themselves to covalent and non-covalent coupling reactions. Thereby, emphasizing on the importance of nanoconjugate structure between rGO and peptides and its remarkable impact on the antimicrobial and hemolytic properties. WCA study revealed a decrease in hydrophobic behaviour of rGO making it hydrophilic upon conjugation with peptide molecules which is an essential characteristic for utilization of conjugate in therapeutic applications.

Enhanced antibacterial activity against *E. coli* along with decrease in hemolysis was exhibited by covalently conjugated peptide-rGO nano bioconjugate. The reported bioengineered nanoconjugate aims to open new avenues for diverse applications such as prospective substitutes to conventional antibacterial platforms being used in sensing to drug delivery scaffolds. Further, it may find its future as an antibiofouling membrane in biomedical devices. This type of peptide functionalized rGO nanoconjugates can serve as suitable candidate for theranostic based applications with improved activity.

## Methods

No experiments were performed on humans or human tissues. Hemolysis analysis was performed using human blood by taking a written informed consent from the volunteer prior to the commencement of the study as per the declaration of Helsinki, 2013, World Medical Association. The experimental protocols were approved by the Research Monitoring Committee, Panjab University and also, Institutional Biosafety Committee, Panjab University, Chandigarh, India (IBSC/PU/2019/158).

### Materials

Graphite Nanopowder (purity 98%), Potassium Permanganate (KMnO_4_) and L-ascorbic acid (C_6_H_8_O_6_) were purchased from Sisco Research Laboratories (SRL) India. Cross linkers: N-Hydroxysuccinimide (NHS) and N-Ethyl-N′-(3-dimethylaminopropyl) carbodiimide (EDC) were purchased from Avra Synthesis, India. De-ionized water (DI) was used in the preparation of samples and other solutions. Chemicals used during the course of this study were of commercial grade and used without further purification. Institutional ethical protocols were followed while performing experiments.

### Apparatus

X-ray Diffraction (XRD) measurements were carried out using X’Pert Pro XRD equipped with x’Celerator solid-state detector. Fourier-Transform Infrared Spectroscopy (FT-IR) studies were performed using Perkin Elmer - Spectrum RX-FT-IR by casting sample on potassium bromide (KBr) pellets within a scan range of 4000–400 cm^−1^. KRÜSS-DSA25E was used to evaluate hydrophilic/phobic behaviour of nanoparticle membranes by measuring static contact angle of a water droplet at room temperature (37 °C). Transmission Electron Microscopy (TEM) was used to study the morphology by using TEM, Hitachi (H-7500) 120 kV. The samples were prepared using the drop casting method. Minimum Inhibitory Concentration (MIC) was evaluated by observing readings using ELISA plate reader.

### Green synthesis of reduced Graphene Oxide

Reduced graphene oxide was prepared using green approach reported previously in the literature^23^. Initially, 1 g of graphite nanopowder was introduced to 50 mL concentrated H_2_SO_4_ followed by constant stirring in a bath containing ice cold water. 3 g KMnO_4_ was slowly added to the mixture by maintaining the temperature below 10 °C. The obtained suspension was stirred using a magnetic stirrer for 25 minutes at 37 °C followed by 5 minutes sonication treatment in an ultrasonic water bath. The stirring-sonication process was repeated several times. 200 mL distilled water was added to the solution to stop the reaction. The pH of the obtained solution was adjusted to ~6 by addition of 1 M NaOH. Reducing solution was prepared by dissolving 10 g L-ascorbic acid in 100 mL distilled water. The solution was added to the exfoliated graphite oxide suspension slowly. Reduction was performed by heating the solution at 95 °C for 1 hour. After the reduction process was over, black precipitates were obtained by filtering the solution through cellulose filter paper. The precipitates were consecutively washed with 1 M HCl solution and distilled water to obtain a neutral pH^47^. Fine rGO powder was obtained by freeze-drying the filtrate for 2–3 days.

### Synthesis of peptide functionalized nano bioconjugate via different interaction mechanisms

Peptide was covalently conjugated with rGO by a method reported previously^48^. In this study, covalently conjugated rGO-peptide complex is abbreviated as rGO-P_C._ In a typical protocol, powdered rGO was dissolved in de-ionized water followed by sonication for 30 minutes to obtain a clear solution of rGO. Cross-linkers, 1-ethyl-3-(3-dimethylaminopropyl) carbodiimide hydrochloride (EDC) and N-Hydroxysuccinimide (NHS) were successively added into the rGO solution and sonicated for 1 hour to generate semi-stable NHS esters linkage with rGO. Then, pH of the mixture was adjusted to 8. The peptides (dissolved in phosphate buffer solution (PBS), 0.145 mM, 0.1 mL) were added to the obtained mixture followed by incubation at 4 °C in dark for 12 hours to enable cross-linking of the NHS esters with peptides. Unbound peptides were removed from the final solution by centrifugation at 11,750 g for 30 minutes. The obtained sediment was then re-dispersed in de-ionized water and centrifuged several times to obtain a homogenous solution.

Whereas, the non-covalent interaction between peptide and rGO complex, abbreviated as rGO-P_S_ was obtained by using a protocol described earlier^49^. Peptide was mixed with rGO suspension in 9:1 ratio (v/v) in PBS followed by incubation at 25 °C for 5 minutes.

### Calculation for number of peptides molecules bound with a nanoparticle

The number of peptide molecules binding with one rGO nanoparticle was obtained by the following equation (Eq. 1)^50^.

1 $${{\rm{N}}}_{{\rm{pep}}}=0.65({{\rm{R}}}_{{\rm{rGO}}\mbox{--}{\rm{pep}}}^{3}\,\mbox{--}\,{{\rm{R}}}_{{\rm{rGO}}}^{3})/{{\rm{R}}}_{{\rm{pep}}}^{3}$$

where,

R_rGO–pep_ = Radius of conjugated nanoparticle

R_rGO_ = Radius of rGO

R_pep_ = diameter of peptide

### Hemolysis assay

The hemolytic assay of rGO and peptide functionalized conjugates (rGO-P_C_, rGO-P_S_) was performed according to the protocol reported previously in literature^46^. Briefly, fresh Ethylenediaminetetraacetic acid (EDTA)-stabilized human whole blood samples were collected from a healthy volunteer. Whole blood was added to PBS followed by centrifugation to isolate Red Blood Cells (RBCs) from serum. Separated RBCs were washed 4 times using PBS at 500 g for 10 minutes at 5 °C. The washed RBCs were resuspended in PBS and stored at low temperature till further use. To test the hemolytic activity of rGO and nano bioconjugates samples, diluted RBC suspension was added to rGO and conjugate suspension solutions in PBS at different concentrations (0–500 μg mL^–1^). DI water (+RBCs) and PBS (+RBCs) were used as the positive control and negative control, respectively. All the samples were placed on a rocking shaker in an incubator at 37 °C for 3 hours. After incubation, the samples were centrifuged at 10,016 g for 3 minutes. Following the literature described protocol^46^, absorbance of hemoglobin in the supernatant was measured at 540 nm using a spectrophotometer. Percent hemolysis was calculated using Eq. 2.

2 $${\rm{Percent}}\,{\rm{haemolysis}}( \% )=\left(\frac{sample\,abs\,540\mbox{--}655\,nm-negative\,control\,abs\,540\mbox{--}655\,nm}{positive\,control\,abs\,540\mbox{--}655\,nm-negative\,control\,abs\,540\mbox{--}655\,nm}\right)\times 100$$

### Minimum inhibitory concentration determination

Microtiter broth dilution method was used to determine the MIC as previously described^32^. MIC is defined as the value of minimal concentration that inhibits visible growth of microorganisms. *E. coli* was cultured according to the ATCC protocols/specifications. The bacterial culture was inoculated in Luria-Bertani (LB) broth and was left to grow for 12 hours at 37 °C and harvested in the mid-exponential growth phase to obtain a final concentration of 10^5^ colony forming units (cfu)/mL followed by centrifugation. The obtained pellet of cells was washed three times and re-suspended in PBS to remove residual macromolecules and other constituents. Then, growth of *E. coli* was monitored spectrophotometrically by measuring the absorbance at 600 nm. Bacterial cell suspension was diluted up to the desired concentration of 10^5^ cfu/mL to bring the desired initial optical density. For MIC protocol, in each well of 96-well plates containing serially diluted samples of rGO, AMP, rGO-P_S_ and rGO-P_C_ were inoculated with bacterial suspension. To avoid any contamination, all the experimental work was done under laminar hood. Then, the samples were allowed to mix with LB media by using shaker at 37 °C for 3 hours followed by overnight incubation at 37 °C. Absorbance was observed at 600 nm after 16 hours and 24 hours, respectively. Microbial suspension in the absence of rGO-peptide conjugate was used as the negative control, while DI water was treated as a positive control. All experiments were performed in triplicates to avoid errors.

### Well diffusion method

Antibacterial activity of the synthesized rGO, rGO-P_C_ and rGO-P_S_ was determined, using the agar well diffusion assay method^51,52^. Approximately 20 ml of molten and cooled media (Nutrient agar) was poured in sterilized petri dishes. The plates were left overnight at room temperature to check for any contamination to appear. The bacterial test organisms were grown in nutrient broth for 24 hours. A 100 ml nutrient broth culture of each bacterial organism (1 × 10^5^ cfu/ml) was used to prepare bacterial lawns. Three agar wells of 5 mm diameter were prepared. The wells were loaded with 20 µl of the synthesized nanoparticles. The plates containing the bacterial and nanoparticles were incubated at 37 °C. The plates were examined for evidence of zones of inhibition, which appear as a clear area around the wells. The diameter of such zones of inhibition was measured for each sample and expressed in millimetre.

## Data Availability

The authors declare that all available data are present in the manuscript.

## References

[1] Liu J, Cui L, Losic D (2013). Graphene and graphene oxide as new nanocarriers for drug delivery applications. Acta Biomater..

[2] Senapati S, Mahanta AK, Kumar S, Maiti P (2018). Controlled drug delivery vehicles for cancer treatment and their performance. Signal Transduct. Target. Ther..

[3] Joshi S (2020). A review on peptide functionalized graphene derivatives as nanotools for biosensing. Microchim. Acta.

[4] Some S (2014). Cancer Therapy Using Ultrahigh Hydrophobic Drug-Loaded Graphene Derivatives. Sci. Rep..

[5] Wang J (2019). *In vitro* and *in vivo* studies of electroactive reduced graphene oxide-modified nanofiber scaffolds for peripheral nerve regeneration. Acta Biomater..

[6] Park S (2011). Hydrazine-reduction of graphite- and graphene oxide. Carbon N. Y..

[7] Kochmann S, Hirsch T, Wolfbeis OS (2012). Graphenes in chemical sensors and biosensors. TrAC - Trends Anal. Chem..

[8] Sharma V (2017). Synthesis and Characterization of Graphene Oxide (GO) and Reduced Graphene Oxide (rGO) for Gas Sensing Application. Macromol. Symp..

[9] Kumar A, Khandelwal M (2014). Amino acid mediated functionalization and reduction of graphene oxide-synthesis and the formation mechanism of nitrogen-doped graphene. New J. Chem..

[10] Ding H (2015). Reduction of graphene oxide at room temperature with vitamin C for RGO-TiO_2_ photoanodes in dye-sensitized solar cell. Thin Solid Films.

[11] Zhang J (2010). Reduction of graphene oxide via L-ascorbic acid. Chem. Commun..

[12] Bahar AA, Ren D (2013). Antimicrobial peptides. Pharmaceuticals.

[13] Preet S, Pandey SK, Kaur K, Chauhan S, Saini A (2019). Gold nanoparticles assisted co-delivery of nisin and doxorubicin against murine skin cancer. J. Drug Deliv. Sci. Technol..

[14] Sharma R (2019). Exploiting chitosan and gold nanoparticles for antimycobacterial activity of *in silico* identified antimicrobial motif of human neutrophil peptide-1. Sci. Rep..

[15] Cuperus T, Van Dijk A, Matthijs MGR, Veldhuizen EJA, Haagsman HP (2016). Protective effect of *in ovo* treatment with the chicken cathelicidin analog D-CATH-2 against avian pathogenic *E. coli*. Sci. Rep..

[16] Schneider VAF (2016). Imaging the antimicrobial mechanism(s) of cathelicidin-2. Sci. Rep..

[17] van Dijk A (2009). Identification of chicken cathelicidin-2 core elements involved in antibacterial and immunomodulatory activities. Mol. Immunol..

[18] van Dijk A (2009). Chicken heterophils are recruited to the site of *Salmonella* infection and release antibacterial mature Cathelicidin-2 upon stimulation with LPS. Mol. Immunol..

[19] Ligorio C (2019). Graphene oxide containing self-assembling peptide hybrid hydrogels as a potential 3D injectable cell delivery platform for intervertebral disc repair applications. Acta Biomater..

[20] Zou X, Zhang L, Wang Z, Luo Y (2016). Mechanisms of the Antimicrobial Activities of Graphene Materials. J. Am. Chem. Soc..

[21] Liu S (2011). Antibacterial activity of Graphite, Graphite Oxide, Graphene Oxide, and Reduced Graphene Oxide: Membrane and Oxidative Stress. ACS Nano.

[22] Navya Rani M, Ananda S, Rangappa D (2017). Preparation of Reduced Graphene Oxide and Its Antibacterial Properties. Mater. Today Proc..

[23] Abdolhosseinzadeh S, Asgharzadeh H, Kim HS (2015). Fast and fully-scalable synthesis of reduced graphene oxide. Sci. Rep..

[24] Zainuddin MF, Nik Raikhan NH, Othman NH, Abdullah WFH (2018). Synthesis of reduced Graphene Oxide (rGO) using different treatments of Graphene Oxide (GO). IOP Conf. Ser. Mater. Sci. Eng..

[25] Díez N, Śliwak A, Gryglewicz S, Grzyb B, Gryglewicz G (2015). Enhanced reduction of graphene oxide by high-pressure hydrothermal treatment. RSC Adv..

[26] Shukla S (2019). Sustainable Graphene Aerogel as an Ecofriendly Cell Growth Promoter and Highly Efficient Adsorbent for Histamine from Red Wine. ACS Appl. Mater. Interfaces.

[27] Wu X (2013). Biomass-Derived Sponge-like Carbonaceous Hydrogels and Aerogels for supercapacitors. ACS nano.

[28] Pai AR, Nair B (2013). Synthesis of Reduced Graphene Oxide Using Novel Exfoliation Technique and its Characterizations. J. Nano- Electron. Phys..

[29] Eckhart KE, Holt BD, Laurencin MG, Sydlik SA (2019). Covalent Conjugation of Bioactive Peptides to Graphene Oxide for Biomedical Applications. Biomater. Sci..

[30] Abd-Wahab, F., Guthoos, H. F. A. & Wan Salim, W. W. A. Solid-State rGO-PEDOT:PSS Transducing Material for Cost-Effective Enzymatic Sensing. *Biosensors* **9**, (2019). 10.3390/bios9010036 PMC6468658 30832254

[31] Qi Y (2019). Aggregation morphology is a key factor determining protein adsorption on graphene oxide and reduced graphene oxide nanomaterials. Environ. Sci. Nano.

[32] Georgakilas V (2012). Functionalization of Graphene: Covalent and Non-Covalent Approaches, Derivatives and Applications. Chem. Rev..

[33] Pal I (2019). A Peptide-Nanoparticle System with Improved Efficacy against Multidrug Resistant Bacteria. Sci. Rep..

[34] Safarpour M, Khataee A, Vatanpour V (2015). Thin film nanocomposite reverse osmosis membrane modified by reduced graphene oxide/TiO_2_ with improved desalination performance. J. Memb. Sci..

[35] Taherian F, Marcon V, Van Der Vegt NFA, Leroy F (2013). What Is the Contact Angle of Water on Graphene?. Langmuir.

[36] Li Z (2013). Effect of airborne contaminants on the wettability of supported graphene and graphite. Nat. Mater..

[37] Bera B (2018). Wetting of water on graphene nanopowders of different thicknesses. Appl. Phys. Lett..

[38] Shin YJ (2010). Surface-Energy Engineering of Graphene. Langmuir.

[39] Rafiee J (2012). Wetting transparency of graphene. Nat. Mater..

[40] Article, R. Prevalence of Cefepime-Resistant *Escherichia coli* in Iran: A Meta-Analysis (2007–2016). **48**, 603–611 (2019). PMC6500536 31110970

[41] Marr AK, Gooderham WJ, Hancock RE (2006). Antibacterial peptides for therapeutic use: obstacles and realistic outlook. Curr. Opin. Pharmacol..

[42] Yacoub, H. A. *et al*. Antimicrobial activities of chicken β-defensin (4 and 10) peptides against pathogenic bacteria and fungi. *Front. Cell. Infect. Microbiol*. **5** (2015). 10.3389/fcimb.2015.00036 PMC4400880 25941665

[43] Zamani E (2019). Mechanistic Understanding of the Interactions of Cationic Conjugated Oligo- and Polyelectrolytes with Wild-type and Ampicillin-resistant *Escherichia coli*. Sci. Rep..

[44] Arakha M, Saleem M, Mallick BC, Jha S (2015). The effects of interfacial potential on antimicrobial propensity of ZnO nanoparticle. Sci. Rep..

[45] Pérez-Peinado C (2018). Mechanisms of bacterial membrane permeabilization by crotalicidin (Ctn) and its fragment Ctn(15–34), antimicrobial peptides from rattlesnake venom. J. Biol. Chem..

[46] Yang J, Gunasekaran S (2013). Electrochemically reduced graphene oxide sheets for use in high performance supercapacitors. Carbon N. Y..

[47] Liao KH, Lin YS, MacOsko CW, Haynes CL (2011). Cytotoxicity of graphene oxide and graphene in human erythrocytes and skin fibroblasts. ACS Appl. Mater. Interfaces.

[48] Akhavan O (2010). The effect of heat treatment on formation of graphene thin films from graphene oxide nanosheets. Carbon N. Y..

[49] Drewniak, S. *et al*. Studies of Reduced Graphene Oxide and Graphite Oxide in the Aspect of Their Possible Application in Gas Sensors. *Sensors (Switzerland)* **16** (2016). 10.3390/s16010103 PMC4732136 26784198

[50] Wang H (2011). Graphene oxide-peptide conjugate as an intracellular protease sensor for caspase-3 activation imaging in live cells. Angew. Chemie - Int. Ed..

[51] Calzolai L, Franchini F, Gilliland D, Rossi F (2010). Protein-Nanoparticle Interaction: Identification of the Ubiquitin-Gold Nanoparticle Interaction Site. Nano Lett..

[52] Satish B, Venkateswara RK, Shilpa CC, Tejaswi T (2013). Synthesisand characterization of graphene oxide and its antimicrobial activity against *Klebseilla* and *Staphylococus*. Int. J. Adv.Biotechnol. Res..

